# Avian strains of emerging pathogen *Escherichia fergusonii* are phylogenetically diverse and harbor the greatest AMR dissemination potential among different sources: Comparative genomic evidence

**DOI:** 10.3389/fmicb.2022.1080677

**Published:** 2023-01-20

**Authors:** Kandhan Srinivas, Sandeep Ghatak, Daniel Aibor Pyngrope, Madesh Angappan, Arockiasamy Arun Prince Milton, Samir Das, Vanita Lyngdoh, John Pynhun Lamare, Mosuri Chendu Bharat Prasad, Arnab Sen

**Affiliations:** ^1^Division of Veterinary Public Health, ICAR – Indian Veterinary Research Institute, Bareilly, India; ^2^Division of Animal and Fisheries Sciences, ICAR Research Complex for North Eastern Hill Region, Umiam, India

**Keywords:** *Escherichia fergusonii*, avian, emerging, phylogeny, AMR, comparative genomics, mobilome, pangenomics

## Abstract

**Introduction:**

*Escherichia fergusonii* is regarded as an emerging pathogen with zoonotic potential. In the current study, we undertook source-wise comparative genomic analyses (resistome, virulome, mobilome and pangenome) to understand the antimicrobial resistance, virulence, mobile genetic elements and phylogenetic diversity of *E. fergusonii*.

**Methods:**

Six *E. fergusonii* strains (5 multidrug resistant strains and 1 biofilm former) were isolated from poultry (duck faeces and retail chicken samples). Following confirmation by phenotypic and molecular methods, the isolates were further characterized and their genomes were sequenced. Comparative resisto-virulo-mobilome analyses and pangenomics were performed for *E. fergusonii* genomes, while including 125 other *E. fergusonii* genomes available from NCBI database.

**Results and discussion:**

Avian and porcine strains of *E. fergusonii* were found to carry significantly higher number of antimicrobial resistance genes (*p* < 0.05) and mobile genetic elements (plasmids, transposons and integrons) (*p* < 0.05), while the pathogenic potential of bovine strains was significantly higher compared to other strains (*p* < 0.05). Pan-genome development trends indicated open pan-genome for all strains (0 < γ < 1). Genomic diversity of avian strains was found to be greater than that from other sources. Phylogenetic analysis revealed close clustering among isolates of similar isolation source and geographical location. Indian isolates of *E. fergusonii* clustered closely with those from Chinese and a singleton Australian isolate. Overall, being the first pangenomic study on *E. fergusonii*, our analysis provided important cues on genomic features of the emerging pathogen *E. fergusonii* while highlighting the potential role of avian strains in dissemination of AMR.

## 1. Introduction

*Escherichia fergusonii* comprises a collection of Gram-negative, rod-shaped facultatively anaerobic, and non-spore-forming bacteria that are oxidase negative, catalase positive, and mostly motile due to peritrichous flagella (Scheutz and Strockbine, 2015). The organism has been regarded as an emerging pathogen with zoonotic significance (Saha et al., 2021) owing to increasing reports of disease conditions ranging from wound infection (Mahapatra et al., 2005) to hemolytic uremic syndrome (HUS) (Baek et al., 2019; Hwang et al., 2021).

Since its recognition as a unique species under the genus *Escherichia* in 1985 (Farmer et al., 1985),^1^ *E. fergusonii* has been identified from various clinical and non-clinical sources. In humans, the organisms have been implicated in cases of wound infections (Funke et al., 1993; Mahapatra et al., 2005; Adesina et al., 2019), diarrhea (Chaudhury et al., 1999; Toualbia et al., 2018), endophthalmitis (Gokhale et al., 2014), cystitis (Savini et al., 2008; Lagacé-Wiens et al., 2010), bacteremia (Lai et al., 2011), urinary tract infection (Bours et al., 2010), and hemolytic uremic syndrome (Baek et al., 2019; Hwang et al., 2021). Besides clinical conditions, the organisms were also isolated from the feces of a male patient with leukemia in Italy (Savini et al., 2009). Among non-human sources, *E. fergusonii* has commonly been identified from avian species including wild and migratory birds (Ben Yahia et al., 2020; Shah et al., 2022), broilers (Forgetta et al., 2012; Oh et al., 2012; Simmons et al., 2016; Pontes et al., 2021; Saha et al., 2021), ducks (Tang et al., 2022), and a case of fibro-necrotic typhlitis in ostrich (Herráez et al., 2005). In other animals, *E. fergusonii* has been identified from diarrheic caprines (Hariharan, 2009) and equines (Weiss et al., 2011), as well as from cases of pneumonia in bovines (Rimoldi and Moeller, 2013). Apart from clinical cases, *E. fergusonii* has also been reported in pigs (Rayamajhi et al., 2011; Guan et al., 2022), sheep, and cattle (Wragg et al., 2009; Parin et al., 2018; Tang et al., 2022). In addition to domestic livestock and birds, contaminations of foods of animal origin by *E. fergusonii* were also reported by many researchers, also emphasizing the food-borne importance of this organism (Silveira et al., 1999; Kola et al., 2012; Maifreni et al., 2013).

Though the organism has been reported from various parts of the world, including Asia (Tang et al., 2022), Europe (Toualbia et al., 2018), North and South Americas (Lindsey et al., 2017; Pontes et al., 2021), and Africa (Ben Yahia et al., 2020), they have hardly been reported from India, and the current report, to the best of our knowledge, is the first comprehensive genomic study from India.

Besides the emerging nature of the pathogen, the organism has also been regarded as a reservoir of antimicrobial resistance (AMR) (Tang et al., 2022). Concerns have been raised about increasing multidrug resistance among *E. fergusonii* strains (Savini et al., 2008; Lagacé-Wiens et al., 2010; Rayamajhi et al., 2011). The presence of AMR genes of clinical importance, such as extended spectrum beta-lactamases (ESBLs) (Lagacé-Wiens et al., 2010), carbapenemases (Ben Yahia et al., 2020), and mobilized colistin resistance (*mcr*) genes (Adesina et al., 2019; Pontes et al., 2021; Tang et al., 2022) among *E. fergusonii* strains has earned global attention. In addition to harboring AMR genes, previous reports have suggested that the organism can possess multiple virulence factors, such as heat-labile toxin (LT), heat-stable toxin (STa), as well as *eae* gene (Rayamajhi et al., 2011; Oh et al., 2012). Other virulence genes reported from *E. fergusonii* included *iss, prfB*, and *ireA*, which were known to be part of the *E. coli* virulence array (Wragg et al., 2009).

Recent advancements in genome sequencing have greatly facilitated the genomic studies of the organisms, enabling a deeper understanding of the organismal biology, functions, genome structure (pan-genome, core-genome, etc.), and epidemiology (Gaiarsa et al., 2015; Caputo et al., 2019; San et al., 2020; Hirabayashi et al., 2021). However, comprehensive genomic studies on *E. fergusonii*, despite escalating the importance of the organism, are lacking. Previously, *E. fergusonii* had been included as either an out-group or as a member of the *Escherichia* genus in various genomic studies pertaining to *E. coli* (Touchon et al., 2009; Lukjancenko et al., 2010; Pedersen, 2017; Yang et al., 2019; Murray et al., 2021).

Therefore, considering the emerging nature of the pathogen and the paucity of comprehensive data on genomic features, we undertook the present study to explore and elucidate the pan-genome, resistome, virulome, and mobilome of *E. fergusonii*while assessing the present and future threats posed by the organism as an emerging food-borne zoonotic pathogen.

## 2. Materials and methods

### 2.1. Isolation and identification of *E. fergusonii*

Isolation of *E. fergusonii* from food (retail chicken and intestine) and animal samples (fecal samples) was undertaken by methods prescribed earlier (Tang et al., 2022). Briefly, the samples were enriched in Luria Broth (HiMedia, India) followed by inoculation onto Simmons Citrate Agar (SCA; HiMedia) supplemented with 2% adonitol (Sisco Research Laboratories, India) (Foster et al., 2010). Dark yellow to orange colonies were presumptively identified as *E. fergusonii*, which were further sub-cultured onto Sorbitol MacConkey agar (SMAC; HiMedia, India) (Tang et al., 2022). Pale or colorless colonies from SMAC plates were subjected to biochemical tests (fermentation of cellobiose and arabinose obtained from HiMedia, India) (Glover et al., 2017). Molecular confirmations of the isolates were done with the help of a uniplex PCR targeting palmitoleoyl–acyl carrier protein (ACP)-dependent acyltransferase (Lindsey et al., 2017).

### 2.2. Phenotypic characterization of isolates

Phenotypic characterization of the isolates involved the evaluation of antibiotic susceptibility and biofilm-forming ability on microtiter plates. Antibiotic susceptibility testing was done for nine antibiotics including ampicillin (10 μg), cefotaxime (30 μg), cefoxitin (30 μg), ceftazidime (30 μg), ciprofloxacin (5 μg), co-trimoxazole (25 μg), gentamicin (10 μg), imipenem (10 μg), and tetracycline (10 μg) following Kirby–Bauer method (Bauer et al., 1966), and interpretation was done as per CLSI guidelines recommended for Enterobacteriaceae (CLSI, 2016). Biofilm-forming abilities of the *E. fergusonii* isolates were estimated as described earlier (Zhang et al., 2016), with modifications in incubation time to 24 h (Naves et al., 2008). *Acinetobacter baumannii* (ATCC 19606) and *Escherichia coli* DH5α were used as the positive and negative controls, respectively (Zhang et al., 2016).

### 2.3. Genome sequencing and assembly

Genomic DNA was extracted using QIAGEN DNA Mini Kit and subjected to whole-genome sequencing on the Illumina platform. The sequence data were checked for quality using the FastQC tool^2^ with default settings, and all sequences passed the quality check as per code developers’ guidelines. Following a quality check, the genomes were assembled using the Shovill tool ver. 1.0.9^3^ with SKESA assembler v. 1.0.9 (Souvorov et al., 2018) and “—trim” switch on (for adaptor trimming) along with “read error” and “post-assembly correction” enabled (default). Species identification of assembled genomes was performed using Kraken2 v.2.0.7 (Wood et al., 2019). Genome sequences were submitted to NCBI under the accession numbers JANDMD000000000, JANDMC000000000, JANDMB000000000, JANDMA000000000, JANDLZ000000000, and JANDLY000000000.

### 2.4. Public genome dataset of *E. fergusonii*

*Escherichia fergusonii* genomes (*n* = 127) submitted to the NCBI genome database up to 11 April 2022 were downloaded for this study. The genomes were found to represent different isolation sources such as human, avian, bovine, ovine, porcine, and environment. One duplicate entry (CP070954.1) for the strain EF44 was found and was therefore excluded. The genomes were coded based on their source and their strain names for ease of analysis. We assigned the codes to each genome based on their isolation source in the format X_Y, where “X” indicated the isolation source and “Y” indicated the strain name. The details of the genomes and corresponding metadata are furnished in Supplementary Table 1. To assess the taxonomic affiliation, downloaded genomes were assessed for their average nucleotide identities (ANI) with a cut-off of 95–96%, as suggested by Richter and Rosselló-Móra (2009) using PYANI v.0.2.12 (Pritchard et al., 2016). Following ANI analysis, the genome of *E. fergusonii* strain Bg39 (NZ_CABHNF010000001.1) was excluded from further analysis. Thus, the working data set comprised 131 genomes (127 downloaded genomes and 6 genomes sequenced in this study minus 2 erroneous genomes).

### 2.5. Resistome analysis

The resistome analyses of *E. fergusonii* genomes were undertaken with the help of the Resistance Gene Identifier (RGI) tool v5.2.1 to predict mutations as well as acquired resistance genes (Alcock et al., 2020). The analysis parameters for RGI runs were set at “contigs,” “perfect and strict hits,” and “include nudge” along with other default settings. The pan- and core-resistomes were constructed with the help of the PRAP tool (He et al., 2020). The pan- and core-resistome curves were fitted following the power law model as described earlier (Tettelin et al., 2008).

### 2.6. Analysis of virulome and pathogenic potential

The virulome analyses of *E. fergusonii* genomes were performed using the ABRicate tool v1.0.1^4^ using the VFDB database updated till 02 August 2022. The criteria for identifying virulence gene(s) were a minimum of 75% sequence similarity with a minimum of 80% coverage. A genome-wide association study using the Scoary tool (Brynildsrud et al., 2016) was undertaken to identify the host-associated virulence factors. Furthermore, the pathogenic potentials of *E. fergusonii* toward humans were assessed using the PathogenFinder v1.1 web tool (Cosentino et al., 2013) with default settings.

### 2.7. Mobilome (plasmids, transposons, and integrons) analyses

*Escherichia fergusonii* genomes (*n* = 131) were searched for the presence of plasmids with the aid of the ABRicate tool v1.0.1 using the PlasmidFinder database updated till 10 May 2022 (see text footnote 4). Transposons and integrons among the *E. fergusonii* genomes were identified with the BacAnt v.3.3.1 tool deploying TransposonDB v.2.0 and IntegronDB v.2.0 (both databases updated till 2021.05.11), respectively (Hua et al., 2021).^5^ The minimum sequence identity threshold used for detecting transposons and integrons was 90%, while the minimum coverage threshold was set at 80%.

### 2.8. Pangenomics of *E. fergusonii*

The pan-genome analysis was undertaken with the help of the Phylogeny Enhanced Pipeline for pan-genome (PEPPAN) tool v1.0.5 (Zhou et al., 2020). To avoid annotation bias, all genomes were re-annotated with the Prokka tool v1.14.5 (Seemann, 2014), and the output files were fed as input for downstream analyses. Trends in pan-genome and core-genome development were estimated using the power law functions as described previously (Tettelin et al., 2008; Zhou et al., 2020).

### 2.9. Phylogenetic analysis

To ascertain the phylogenetic relationship among the *E. fergusonii* isolates, genomes were aligned with the PRANK module^6^ of the Roary v.3.13.0 tool (Page et al., 2015) and were curated with the GBlocks v0.91b tool to remove inaccurately aligned regions (Castresana, 2000). In both runs, the default settings of the tools were used. Furthermore, the phylogenetic tree construction was done with the help of the IQ-TREE v1.6.12 software (Nguyen et al., 2015) run with the ModelFinder (Kalyaanamoorthy et al., 2017) and was visualized using the FigTree v.1.4.4 tool.^7^

### 2.10. Statistical analysis and data representation

Data collation and analyses were done with the help of MS-Excel^R^ and the IBM SPSS Statistics Subscription Build 1.0.0.1447. Data visualization was facilitated using R Studio v.2022.02.3 Build 492. The Venn diagrams for graphical representation were created with the help of the VennPainter v1.2.0 tool (Lin et al., 2016).

## 3. Results

### 3.1. Antimicrobial susceptibility and biofilm formation by *E. fergusonii* isolates

**FIGURE 1:**
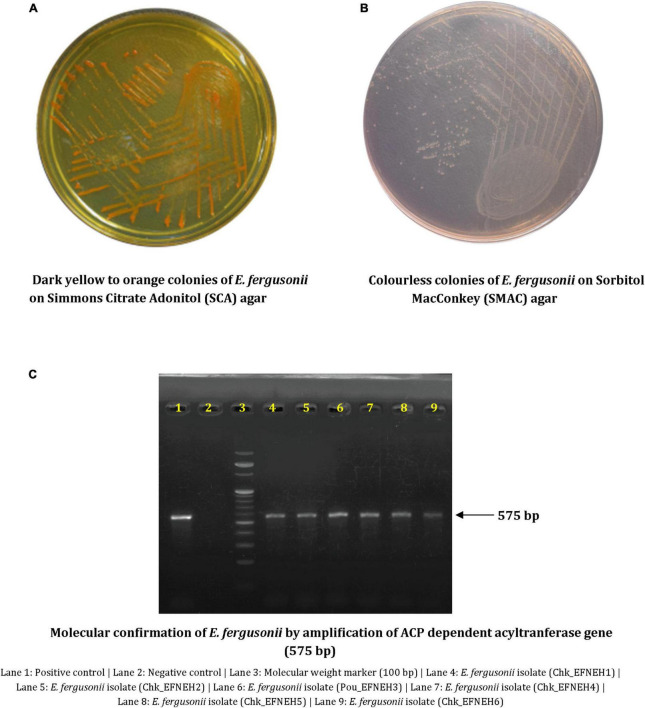
Phenotypic and molecular characterization of *Escherichia fergusonii* isolates. **(A)** Dark yellow to orange colonies of *E. fergusonii* on Simmons Citrate Adonitol (SCA) agar. **(B)** Colorless colonies of *E. fergusonii* on Sorbitol MacConkey (SMAC) agar. **(C)** Molecular confirmation of *E. fergusonii* by amplification of ACP dependent acyltransferase gene (575 bp).

*Escherichia fergusonii* isolates (*n* = 6) were recovered from samples (*n* = 48) of duck feces (*n* = 4) and retail chickens (*n* = 44), indicating an overall occurrence of 12.5% (6/48) among the samples. These isolates showed dark yellow to orange colonies on SCA supplemented with 2% adonitol and were colorless on SMAC (Figures 1A, B). All isolates yielded positive reactions for cellobiose and arabinose fermentation (Table 1). Molecular confirmation by PCR was based on the positive amplification of palmitoleoyl–ACP-dependent acyltransferase gene with a 575-bp product size (Figure 1C).

**TABLE 1:** Phenotypic and genotypic characters of *E. fergusonii* isolated and sequenced.

Isolates	Sugar fermentation		AST phenotype*									Biofilm formation	Integrons	Plasmids in genome	Transposon types	Pathogenic potential**
			**AMP**	**GEN**	**CIP**	**TE**	**COT**	**CTX**	**FOX**	**IPM**	**CAZ**					
	**Arabinose**	**Cellobiose**	**10**	**10**	**5**	**30**	**25**	**30**	**30**	**10**	**30**					
Chk_EFNEH1	Positive	Positive	22 S	16 S	15 R	10 R	6 R	16 R	23 S	25 S	21 S	Negative	Yes	4	130	0.933
Chk_EFNEH2	Positive	Positive	21 S	16 S	15 R	10 R	6 R	18 R	23 S	25 S	23 S	Negative	Yes	4	130	0.933
Pou_EFNEH3	Positive	Positive	20 S	15 S	26 S	24 S	21 S	15 R	20 S	25 S	18 I	Negative	No	1	39	0.939
Chk_EFNEH4	Positive	Positive	22 S	15 S	18 R	13 I	6 R	15 R	24 S	27 S	20 I	Negative	Yes	1	117	0.934
Chk_EFNEH5	Positive	Positive	6 R	15 S	15 R	6 R	20 S	18 R	24 S	27 S	22 S	Negative	No	10	132	0.923
Chk_EFNEH6	Positive	Positive	6 R	15 S	19 R	11 R	6 R	15 R	25 S	26 S	21 S	Positive	Yes	3	136	0.930

*Disc concentrations in mcg; S, sensitive; I, intermediate; R, resistant. AMP, ampicillin; GEN, gentamicin; CIP, ciprofloxacin; TE, tetracycline; COT, Co-trimoxazole; CTX, cefotaxime; FOX, cefoxitin; IPM, imipenem; CAZ, ceftazidime.

**As per Cosentino et al. (2013).

Antibiotic susceptibility testing revealed that all 6 isolates were resistant to cefotaxime, followed by ciprofloxacin, tetracycline, and co-trimoxazole (4/6), whereas none of the isolates were resistant to cefoxitin, imipenem, ceftazidime, and gentamicin. Multidrug resistance was exhibited by 4 (Chk_EFNEH1, Chk_EFNEH2, Chk_EFNEH5, and Chk_EFNEH6) of the 6 isolates (Table 1). Crystal-violet-stained microtiter plates for the quantification of the biofilm formation revealed that only Chk_EFNEH6 isolate was positive for biofilm formation (Table 1).

### 3.2. Genome features

*Escherichia fergusonii* genomes (*n* = 131) included in this study ranged from 4.20966 to 5.18638 Mb in size with a mean size of 4.78396 ± 0.01682 Mb (4.75067, 4.81723). The year of isolation of the genomes ranged from 1983 (strain MOD1-EC5837) to 2022 (strains EFNEH3 to EFNEH6). The majority of the genomes were of avian origin (*n* = 57), followed by ovine (*n* = 25), porcine (*n* = 22), unknown (*n* = 12), bovine (*n* = 11), human (*n* = 3), and environmental origin (*n* = 1). Genomes from human, environmental, and unknown origin were excluded from statistical analyses owing to the lesser number of genomes. The quality assessment of genomes returned satisfactory results. Estimation of the ANI revealed a percentage value well above the cut-off of 95–96% except for one genome (Bg39; NZ_CABHNF010000001.1), and thus, it was excluded for further analyses (Supplementary Figure 1).

### 3.3. Resistome analysis

**FIGURE 2:**
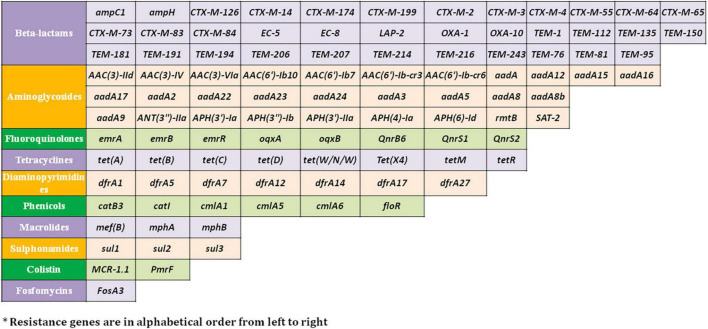
Resistance genes* against major antimicrobial classes identified in the *E. fergusonii* genomes.

**FIGURE 3:**
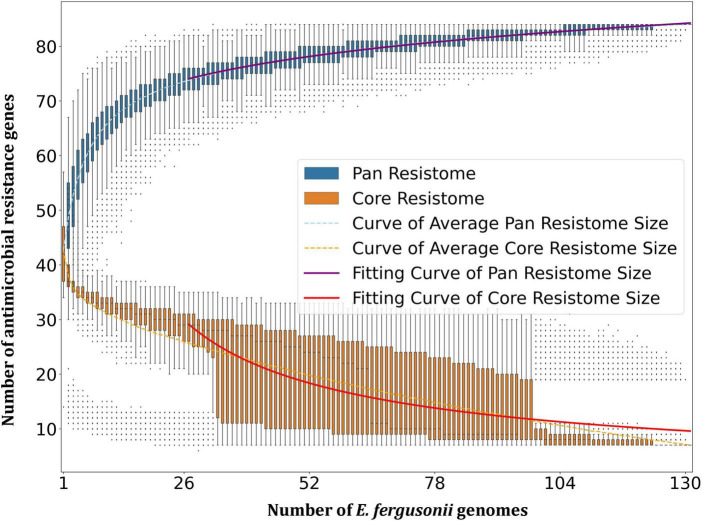
Pan-resistome and core-resistome development trends of *E. fergusonii* genomes.

Resistome prediction by CARD revealed the presence of 152 different varieties of antibiotic resistance ontologies (AROs) among the 131 genomes (Supplementary Table 2). All the genomes of *E. fergusonii* carried 15 AROs, namely *acrB, bacA, cpxA*, CRP, *emrA, Escherichia coli acrA, Escherichia coli* AcrAB-TolC, with AcrR mutation conferring resistance to ciprofloxacin, tetracycline, and ceftazidime; *Escherichia coli* soxR with mutation conferring antibiotic resistance; *Escherichia coli* soxS with mutation conferring antibiotic resistance; H-NS, *msbA, qacJ, rsmA, TolC*, and *YojI*. Kruskal–Wallis and Bonferroni tests revealed that the isolates of avian and porcine origin carried a significantly higher (*p* < 0.05) number of AMR genes than *E. fergusonii* isolates of bovine and ovine origin. The CTX-M variants identified in the genomes were CTX-M-2, CTX-M-3, CTX-M-4, CTX-M-14, CTX-M-55, CTX-M-64, CTX-M-65, CTX-M-73, CTX-M-83, CTX-M-84, CTX-M-126, CTX-M-174, and CTX-M-199. With the exception of CTX-M-65 and CTX-M-174, all other genomes carrying CTX genes were of avian origin only. The OXA variants identified in the genomes of *E. fergusonii* included OXA-1 and OXA-10, and both were identified only in avian isolates. Tet(X4), responsible for tigecycline resistance, was identified in two isolates of avian origin (Duck_YC114-2 and Chk_HNCF11W). The Colistin resistance gene, MCR-1.1, could be detected in isolates of avian origin as well as the singleton isolate from soil (6S41-1). The greatest diversity of AMR genes was observed for beta-lactam and aminoglycoside antibiotics with 35 and 29 genes conferring resistance to these classes of antibiotics, respectively (Figure 2). However, none of our six isolates of *E. fergusonii* carried CTX and MCR variants, though one isolate (Chk_EFNEH5) carried TEM-1 and another of our isolate (Chk_EFNEH6) carried TEM-135. The gamma values of pan-resistome and core-resistome were estimated to be 0.0816 and 0.7033, respectively, indicating the openness of the pan-resistome. The trends in pan-resistome and core-resistome developments were fitted to a power law model with *R*^2^-values of 0.9987 and 0.9349, respectively (Figure 3).

### 3.4. Virulome analysis

A total of 88 virulence genes were identified among the 131 genomes of *E. fergusonii* out of which 25 genes were present in all genomes (Supplementary Table 3). The virulence genes belonged to different virulence factor categories such as adherence, invasion, iron uptake, regulation, secretion system, toxin, anti-phagocytosis, efflux pump, and non-fimbrial adherence determinants. The invasion-associated genes (*ibeB and ibeC*), iron-uptake genes (*entA*, *entB*, *entC*, *entD*, *entE*, *entS*, *fepA*, *fepB*, *fepC*, *fepD*, *fepG*, and *fur*), regulatory genes (*rpoS* and *rcsB*), efflux pump (*acrB*), and miscellaneous genes (*fes* and *gndA*) were identified in all the genomes of *E. fergusonii*. The strain Chk_40A, isolated from the avian source, carried the largest number of virulence genes (59). Among *E. fergusonii* isolated in the present study, two isolates (Pou_EFNEH3 and Chk_EFNEH5) carried the highest number of virulence genes (48). The only toxin identifiable in our analysis was heat-stable enterotoxin-1 (EAST-1) from almost all isolation sources. However, none of the isolates sequenced in this study carried any toxin-associated gene. The genome-wide association study revealed that the virulence genes *cdia*, *lpxD*, *galE_2, galE_3, cdiA*, and *clpB* were found to be significantly associated with the ovine isolates (*p* < 0.05, OR > 90), whereas, the poultry isolates were associated with the virulence genes *iucA, iucB, iucC, iucD, traG, virB1, virB4*, and *virB8* (*p* < 0.05, OR > 40).

Results from the PathogenFinder revealed that all the strains of *E. fergusonii* included in this study were potentially pathogenic to humans with a mean probability of 0.9277 ± 0.0005 (0.9267, 0.9286, 95% CI) (Supplementary Table 4). Kruskal–Wallis and Bonferroni tests revealed that the pathogenic probabilities of the bovine strains of *E. fergusonii* were significantly higher than the strains from other sources (*p* < 0.05). Interestingly, 5 of the 6 isolates sequenced in this study (except Chk_EFNEH5) had probability scores greater than the mean pathogenic probability score of *E. fergusonii*.

### 3.5. Mobilome analysis

**FIGURE 4:**
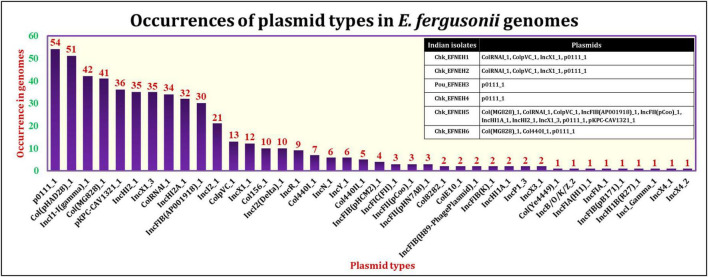
Occurrences of various plasmid types in *E. fergusonii* genomes.

The PlasmidFinder tool identified the occurrences of 40 different plasmid types among the *E. fergusonii* genomes (Figure 4). The most frequently occurring plasmid was p0111_1 (54/131), followed by the plasmids col(pHAD28)_1 (51/131) and IncI1-I(gamma)_1 (42/131). All six isolates from the present study carried the plasmid p0111_1. The isolate Chk_EFNEH5 carried the largest number of plasmids (Col(MG828)_1, ColRNAI_1, ColpVC_1, IncFIB(AP001918)_1, IncFII(pCoo)_1, IncHI1A_1, IncHI2_1, IncX1_3, pKPC-CAV1321_1) in addition to the plasmid p0111_1. On application of the Kruskall–Wallis test, a significant difference was identified among the sources in terms of carriage of plasmids (*p* < 0.05). Subsequently, the Bonferroni test revealed that the avian and the porcine strains of *E. fergusonii* carried a significantly greater number of plasmids (*p* < 0.05).

**FIGURE 5:**
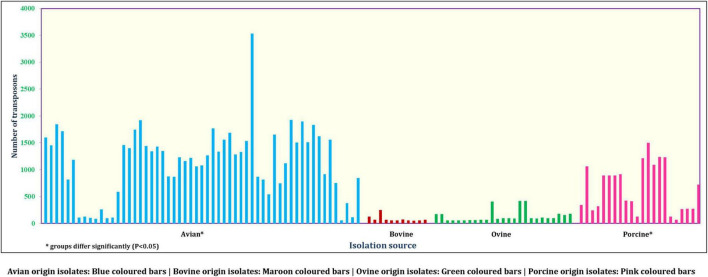
Number of transposons identified in *E. fergusonii* genomes.

**FIGURE 6:**
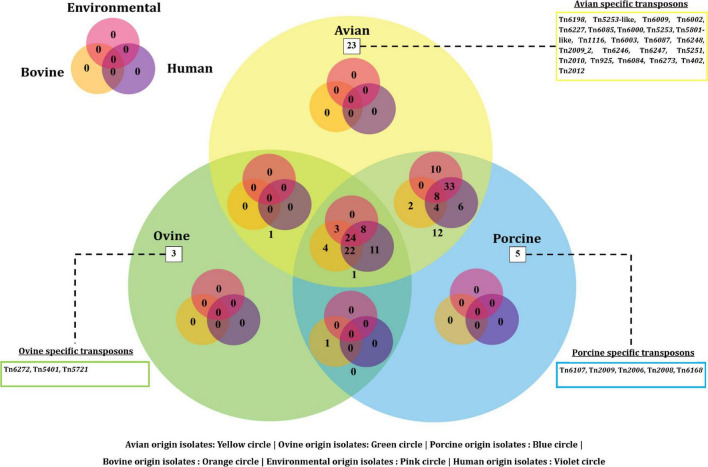
Venn diagram depicting the categorical distribution of various transposon types among *E. fergusonii* genomes.

On the other hand, a total of 190 transposon types were identified among the 131 genomes of *E. fergusonii* (Supplementary Table 5). The common set of transposons observed among all *E. fergusonii* genomes included Tn*6302*, Tn*6183*, Tn*6097*, Tn*5041*-like, Tn*4676*, Tn*6290*, Tn*6291*, Tn*10*, Tn*6177*, Tn*6176*, Tn*4655*, Tn*2610*, Tn*2502*, Tn*6233*, Tn*6019*, Tn*5542*, Tn*6178*, and Tn*6228*. The Kruskal-Wallis and Bonferroni tests revealed a similar trend as that observed for AMR genes, i.e., *E. fergusonii* isolates of avian and porcine origin carried significantly higher (*p* < 0.05) number of transposons compared to the isolates of bovine and ovine origin (Figure 5). The highest number of transposons present in a single isolate was 3,531 which were detected in the isolate Chk_EFCF056. Among the isolates of the present study, however, the common set of transposons comprised 36 different transposon types (Tn*6934*, Tn*9*-like, Tn*6178*, Tn*7051*, Tn*2555.3*, Tn*6286*, Tn*6183*, Tn*2610*, Tn*6214*, Tn*6027*, Tn*6187*, Tn*1722*, Tn*5041*-like, Tn*6233*, Tn*1721*, Tn*4676*, Tn*7*, Tn*7*-like, Tn*6181*, Tn*6301*, Tn*6302*, Tn*5542*, Tn*4655*, Tn*2502*, Tn*1332*, Tn*5422*, Tn*10*, Tn*6097*, Tn*6019*, Tn*6283*, Tn*6228*, Tn*6171*, Tn*6291*, Tn*6290*, Tn*6176*, and Tn*6177*). Interestingly, we observed multiple occurrences of many individual transposable elements in the genomes of *E. fergusonii* isolates from our study (Supplementary Table 6). Overall, the analysis of the transposable elements according to the sources of isolation (Figure 6), revealed that 23 transposon types (Tn*6198*, Tn*5253*-like, Tn*6009*, Tn*6002*, Tn*6227*, Tn*6085*, Tn*6000*, Tn*5253*, Tn*5801*-like, Tn*1116*, Tn*6003*, Tn*6087*, Tn*6248*, Tn*2009_2*, Tn*6246*, Tn*6247*, Tn*5251*, Tn*2010*, Tn*925*, Tn*6084*, Tn*6273*, Tn*402*, and Tn*2012*) were avian specific, 5 (Tn*6107*, Tn*2009*, Tn*2006*, Tn*2008*, and Tn*6168*) were porcine specific, and 3 (Tn*6272*, Tn*5401*, and Tn*5721*) were ovine specific.

**FIGURE 7:**
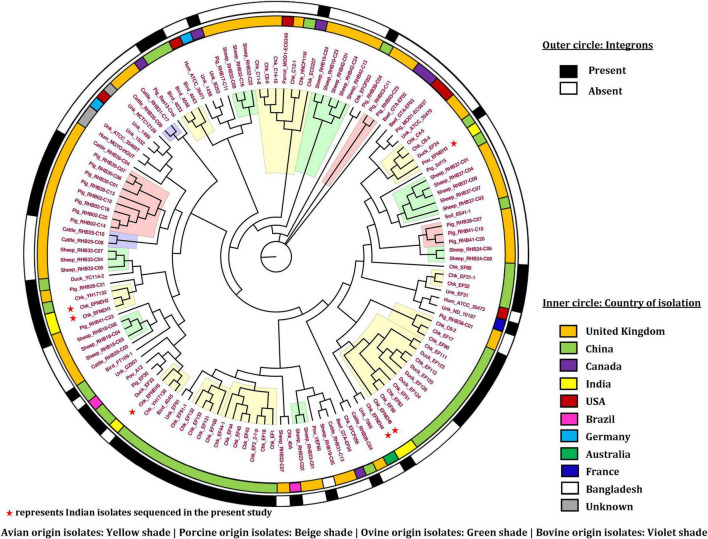
Phylogenetic analysis of *E. fergusonii* genomes included in the study.

A total of 62 *E. fergusonii* genomes were found to harbor integrons (Figure 7). Among the isolates with a known isolation source, only strains of avian and porcine origin carried the integrons. However, four out of six isolates reported in this study harbored integrons. The strains Chk_EFNEH1, Chk_EFNEH2, and Chk_EFNEH4 carried the In498 integron type, whereas, Chk_EFNEH6 carried the In718 integron type, both belonging to the Integron class 1.

### 3.6. Pangenomics

Pan-genome analysis (Supplementary Table 7) revealed that the pan-genome of *E. fergusonii* consisted of 12,764 genes while the core-genome comprised 2,844 genes. When analyzed according to host groups, varying sizes of pan-genomes were revealed for various host groups of *E. fergusonii* (Supplementary Figures 2A–E and Supplementary Table 7). By applying Heaps’ Law of pan-genome development (Tettelin et al., 2008), it was clear that pan-genomes of *E. fergusonii*, as a whole and from various host groups (avian, ovine, porcine, and bovine), were open as the value of γ ranged between 0.144 ± 0.005 and 0.197 ± 0.002, which satisfied the condition for open pan-genome (0 < γ < 1) (Tettelin et al., 2008). Assessment of genome diversities from R_CP_ (ratio of core-genome to pan-genome) values (Ghatak et al., 2016) indicated that *E. fergusonii* strains of avian origin were the most diversified (R_CP_ = 0.33), while those of bovine origin were the most conserved (R_CP_ = 0.54).

### 3.7. Phylogeny

The phylogenetic tree (Figure 7) drawn from the best-fitted model (GTR + F + R6) having the greatest BIC (Bayesian Information Criterion) score revealed clustering of *E. fergusonii* genomes according to the host species. Close clustering of the porcine isolates was identified in three instances (Beige shaded areas, Figure 7). Though the avian isolates formed interspersed clusters, these isolates with the same geographical locations tended together (Yellow shaded areas, Figure 7). These clustering patterns also conformed to the geographical origins of the *E. fergusonii* isolates, especially those of the Chinese and UK origins. These findings indicated the existence of specific *E. fergusonii* lineages associated with their geographical origin as well as the host species. However, the Indian isolates of *E. fergusonii*, which were sequenced in this study, did not form any single cluster and were rather sprinkled over the phylogram. Broadly, the Indian isolates of *E. fergusonii* were associated with the Chinese isolates of similar sources (avian) except the Chk_EFNEH4 strain, which clustered with the Australian isolate (Unk_7966). Human isolates of *E. fergusonii* were associated with the isolates of uncertain origin, and thus, we could not determine direct evidence of possible zoonotic events *via* clonal linkage.

## 4. Discussion

*Escherichia fergusonii* has recently garnered attention in terms of being an emerging pathogen of humans as well as of animals with zoonotic potential (Saha et al., 2021). Food animals serve as an important source, by virtue of which dissemination of AMR through the food chain comes into the picture (Tang et al., 2022). Our study focused on the isolation and assessment of antibiotic susceptibility, virulence, and pathogenic potential, while elucidating genomic features of the genus *E. fergusonii* as a whole through an omics-based approach coupled with a microbiological investigation. We isolated 6 strains of *E. fergusonii* from avian sources (3 from chicken meat, 2 from chicken intestine, and 1 from duck feces), out of which one was a biofilm-forming multidrug resistant strain (Chk_EFNEH6). The biofilm-forming ability of *E. fergusonii* has rarely been explored previously (Ingle et al., 2011). As observed in our study, *E. fergusonii* has also been reported earlier in chicken intestines (Li et al., 2020), duck fecal sources (Tang et al., 2022), and chicken meat (Kola et al., 2012). To the best of our knowledge, this is the first report of isolation and characterization of *E. fergusonii* from a retail meat sample of poultry from India. Identification of *E. fergusonii* in chicken intestines and meat is a matter of public health concern as chicken intestines, besides meat, are consumed in many countries including India (authors’ own observation).^8^

Intensification of the pig and poultry industry has always been in the limelight for the usage of antimicrobial growth promoters (AGP) to increase productivity. However, this has resulted in the emergence of antimicrobial-resistant strains over time (Ben Lagha et al., 2017). Our results of source analysis of the *E. fergusonii* revealed that the avian and porcine origin isolates can potentially serve as important disseminators of AMR owing to their higher carriage rates of transposons, integrons, and AMR genes.

Mobile colistin resistance (MCR) genes are plasmid-borne genetic elements that render colistin ineffective by a phosphoethanolamine-mediated inhibition of the binding of the drug to the cell membrane (Liu et al., 2016). Colistin resistance has been genotypically and phenotypically reported among *E. fergusonii* strains isolated from various sources (Glover et al., 2017; Adesina et al., 2019; Pontes et al., 2021; Lin et al., 2022; Liu et al., 2022; Tang et al., 2022). Tigecycline resistance gene, tet(X4), which too is plasmid-borne, has been reported previously in *E. fegusonii* isolated from pigs (Guan et al., 2022) and poultry (Li et al., 2020). A similar finding was evident in our comparative resistome analysis as well. Nevertheless, it was interesting to note that the colistin resistance gene was identified only in *E. fergusonii* isolates from avian and soil sources, whereas tet(X4), which confers resistance to all tetracyclines including tigecycline, was identified only in isolates from avian sources. These results suggested that poultry could be a major reservoir of AMR *E. fergusonii.* Resistome analysis using RGI/CARD also revealed the presence of ESBL genes such as TEM, SHV, CTX-M, and OXA among the 131 genomes of *E. fergusonii*, which corroborated with earlier reports (Kola et al., 2012; Adesina et al., 2019; Ben Yahia et al., 2020).

In our analysis, we observed that 2 of the 6 isolates (Chk_EFNEH5 and Chk_EFNEH6) phenotyped in the present study were resistant to ampicillin (Table 1). Resistome data (Supplementary Table 2) for these two isolates corroborated well with the phenotypic data as only these two isolates of the six possessed *TEM-1* and *TEM-135* which confer resistance to ampicillin (Alcock et al., 2020). For gentamicin resistance, phenotypic data indicated uniform susceptibility for all 6 Indian isolates, which was supported by the resistome fingerprint of the isolates, revealing the absence of genes conferring gentamicin resistance, though APH(3′)-Ia, APH(6)-Id, and APH(3″)-Ib encoding resistance to streptomycin were observed. Ciprofloxacin susceptibility profiles of the 6 isolates sequenced in this study could be explained by the presence of Qnr variants (*QnrB6* and *QnrS1*) in resistant isolates only. Interestingly, *gyr*A mutation (S83L) conferring resistance to fluoroquinolone was present in 3 isolates (Chk_EFNEH1, Chk_EFNEH2, and Chk_EFNEH5), for which we noticed decreased (19%) zones of inhibition, possibly indicating synergistic effects of multiple mechanisms. However, this needs to be investigated further. Similarly, in the case of tetracycline, we observed reduced susceptibility in all Indian isolates, except Pou_EFNEH3, which tallied well with the presence of the *tet(A*) gene in these isolates, and a possible synergism between *tet(A*) and *tet(B*) was observed for the isolate Chk_EFNEH5 with a 45% reduction in the zone of inhibition. Co-trimoxazole resistance was observed in isolates Chk_EFNEH1, Chk_EFNEH2, Chk_EFNEH4, and Chk_EFNEH6, and resistome data for these 4 isolates revealed the presence of integron-mediated *dfrA14*, possibly mediating resistance to co-trimoxazole. However, we observed the presence of *sul2* gene in some of our isolates. All 6 *E. fergusonii* isolates sequenced in this study exhibited cefotaxime resistance, which was likely due to the presence of EC-8 beta-lactamase in their resistomes. Susceptibilities to cefoxitin and imipenem were observed for all 6 isolates, and correspondingly, we did not detect any genes encoding resistance to these antibiotics. None of the Indian isolates of *E. fergusonii* were resistant to ceftazidime, which matched with the absence of specific genes conferring ceftazidime resistance. However, the generic efflux pump (AcrAB-TolC) effective against multiple antibiotics including ceftazidime could be identified in all isolates, possibly indicating that the presence of such efflux mechanisms may not be determinative of phenotype as was previously highlighted (Cooper et al., 2020). Overall, phenotype and resistome data were in harmony with each other as was previously documented (Hendriksen et al., 2019), and our choice of the method/tool for deciphering resistome appeared reasonable.

The virulence repertoire of *E. fergusonii* is yet to be explored thoroughly (Gaastra et al., 2014). In previous studies, *E. fergusonii* was screened for the presence of *E. coli* virulence genes (Wragg et al., 2009; Ingle et al., 2011). Genes of significant importance such as EAST1 and LT were reported from South Korean pigs (Rayamajhi et al., 2011) and poultry (Oh et al., 2012), respectively, implying the role played by poultry and pigs in harboring virulent *E. fergusonii* organisms. However, six Indian isolates sequenced in our study did not harbor any toxin gene, and similar observations were previously reported by other researchers too (Saha et al., 2021; Lin et al., 2022; Liu et al., 2022). GWAS (Genome-Wide Association Study) analysis indicated host-specific sets of virulence genes implying host adaptation among *E. fergusonii* of avian and ovine origin. However, we could not determine similar sets of genes for other hosts due to insufficient numbers of genomes available from these hosts. These results might pave the way for the future development of virulence-based molecular tools for the detection of *E. fergusonii* in specific hosts. Pathogenic potential analysis of *E. fergusonii* genomes revealed that all *E. fergusonii* were highly likely to be human pathogens, reinforcing the importance of this organism as an emerging pathogen. Interestingly, *E. fergusonii* isolates of bovine origin were found to be comparatively more pathogenic than that from other sources, highlighting the role of food animals as a reservoir of pathogenic *E. fergusonii* as opined earlier also (Tang et al., 2022). Nonetheless, this observation is in contrast with the carriage of AMR genes which were mostly harbored by avian and porcine strains of *E. fergusonii*.

The mobilome of an organism refers to the universal set of mobile genetic elements comprising subsets formed by plasmids, transposons, and integrons. Screening of 131 genomes of *E. fergusonii* for plasmid signatures revealed the predominance of p0111_1 plasmid followed by the plasmid type col(pHAD28)_1. The p0111_1 plasmid was previously reported in association with colistin and tigecycline resistance in *E. fergusonii* (Li et al., 2020; Pontes et al., 2021; Saha et al., 2021; Lin et al., 2022). Even though all *E. fergusonii* genomes sequenced in our study possessed p0111_1 plasmid, they did not harbor genetic elements encoding colistin resistance. A similar trend was observed for the plasmid IncH12, which reportedly was a major carrier of colistin resistance genes in *Salmonella* spp. (Tang et al., 2020). Nevertheless, our results indicated greater occurrences of plasmids among genomes of *E. fergusonii* isolated from avian and porcine sources as was observed for AMR genes. Taken together, these findings implied a greater propensity of dissemination of AMR by *E. fergusonii* of avian and porcine origin.

Transposons are another subset of mobile genetic elements capable of jumping across DNA molecules that often end up disseminating AMR (Babakhani and Oloomi, 2018). Integrons, on the other hand, mediate the dissemination of AMR by integrating genetic elements onto the genomes of the organisms. Of the transposons identified in the genomes of *E. fergusonii*, Tn*5041*-like and Tn*10* were significant (Babakhani and Oloomi, 2018). The transposon Tn*5041*-like variants are non-composite transposons that are usually associated with mercury resistance (Babakhani and Oloomi, 2018), whereas Tn*10* transposons are composite transposons associated with tetracycline resistance (Babakhani and Oloomi, 2018). In the current analysis, host-specific transposons were observed for avian-, porcine-, and ovine-origin *E. fergusonii* isolates, possibly indicating evolutionary adaptation of these strains in the micro-ecosystems connected to their hosts. On the other hand, multiple occurrences of individual transposable elements in the genomes of *E. fergusonii* were perhaps indicative of the highly mobile nature of these elements, thus obscuring their role as possible evolutionary markers for *E. fergusonii*. However, while interpreting data on occurrences of transposons, limitations of the available transposon database and employed bioinformatic tool (BacAnt) should also be considered, including the detection of incomplete transposons based on sequence similarity (80%). Similarly, search for integrons among *E. fergusonii* genomes revealed occurrences of integron elements only in the genomes of *E. fergusonii* of avian and porcine origin. Combined with previous findings of higher occurrences of AMR genes and plasmids in *E. fergusonii* of avian and porcine origin, the current observations reemphasized their potential role in dissemination of AMR to other organisms.

Our analysis revealed open pan-genomes for *E. fergusonii*, indicating the possibility of the pan-genome growing as more and more genomes are sequenced. R_CP_ values are known to be inversely related to the genetic diversity of the strains (Ghatak et al., 2016), and our results indicated that the avian strains had greater genetic diversity followed by the porcine strains, implying a larger fraction of variable genome, possibly contributed by more frequent genetic acquisitions in these strains of *E. fergusonii*. However, pan-genome data needs to be interpreted cautiously as pan-genome size is known to increase with an increment in the number of genomes sequenced (Tettelin et al., 2005; Ghatak et al., 2016). Nevertheless, to the best of our knowledge, our study is the first to analyze and report the pan-genome of the emerging pathogen, *E. fergusonii*.

Phylogenetic analysis of *E. fergusonii* revealed clustering of the genomes according to host species and geographical origin, indicating likely lineages of the organism. This was not uncommon as studies involving other organisms also showed similar trends in clustering (Luo et al., 2021). Our results also revealed that Indian isolates of *E. fergusonii* were associated predominantly with Chinese isolates. Though we could not ascertain the epidemiological link for such observation, the role of migratory birds in the dissemination of *E. fergusonii* had previously been highlighted (Shah et al., 2022). As the Indian subcontinent falls under one of the major flyways of winter migration of birds (Malik et al., 2021), this aspect needs further investigation.

## 5. Conclusion

Taken together, in the present study, we isolated and whole-genome sequenced *E. fergusonii* from poultry (chicken and duck). Furthermore, comparative genomic analysis (pan-genome, resistome, mobilome, virulome, and phylogeny), which, to the best of our knowledge, is the first for *E. fergusonii*, revealed an open pan-genome for *E. fergusonii*, a higher pathogenic potential for the strains of bovine origin, and greater AMR dissemination potential among the strains of avian origin owing to higher carriage rate of mobile genetic elements and AMR genes in the latter group. Phylogenomic analysis, besides revealing greater genomic diversity for avian strains, also elucidated geo-concordant clustering supported by host species of the isolates. Thus, our results assert the importance of avian strains of *E. fergusonii* as potential disseminators of AMR.

## Data availability statement

The datasets presented in this study can be found in online repositories. The names of the repository/repositories and accession number(s) can be found in the article/Supplementary material.

## Author contributions

KS: investigation, formal analysis, visualization, and writing—original draft. SG: conceptualization, methodology, investigation, writing—original draft, and supervision. DP: investigation and formal analysis. MA: investigation, writing—reviewing and editing, and visualization. AM and SD: formal analysis and writing—review and editing. VL: formal analysis. JL: investigation. MP: writing—reviewing and editing. AS: writing—final draft and reviewing and editing. All authors contributed to the article and approved the submitted version.

## Footnotes

1
https://lpsn.dsmz.de/species/escherichia-fergusonii

2
https://www.bioinformatics.babraham.ac.uk/projects/fastqc/

3
https://github.com/tseemann/shovill

4
https://github.com/tseemann/abricate

5
https://github.com/xthua/bacant

6
http://www.wasabiapp.org/software/prank

7
https://github.com/rambaut/figtree/releases

8
https://en.wikipedia.org/wiki/Offal
