# Supplementary figures and images for: Avian strains of emerging pathogen Escherichia fergusonii are phylogenetically diverse and harbor the greatest AMR dissemination potential among different sources: Comparative genomic evidence
Source: Front Microbiol. 2023 Jan 20;13:1080677. doi: 10.3389/fmicb.2022.1080677 (PMC9895846; doi:10.3389/fmicb.2022.1080677)

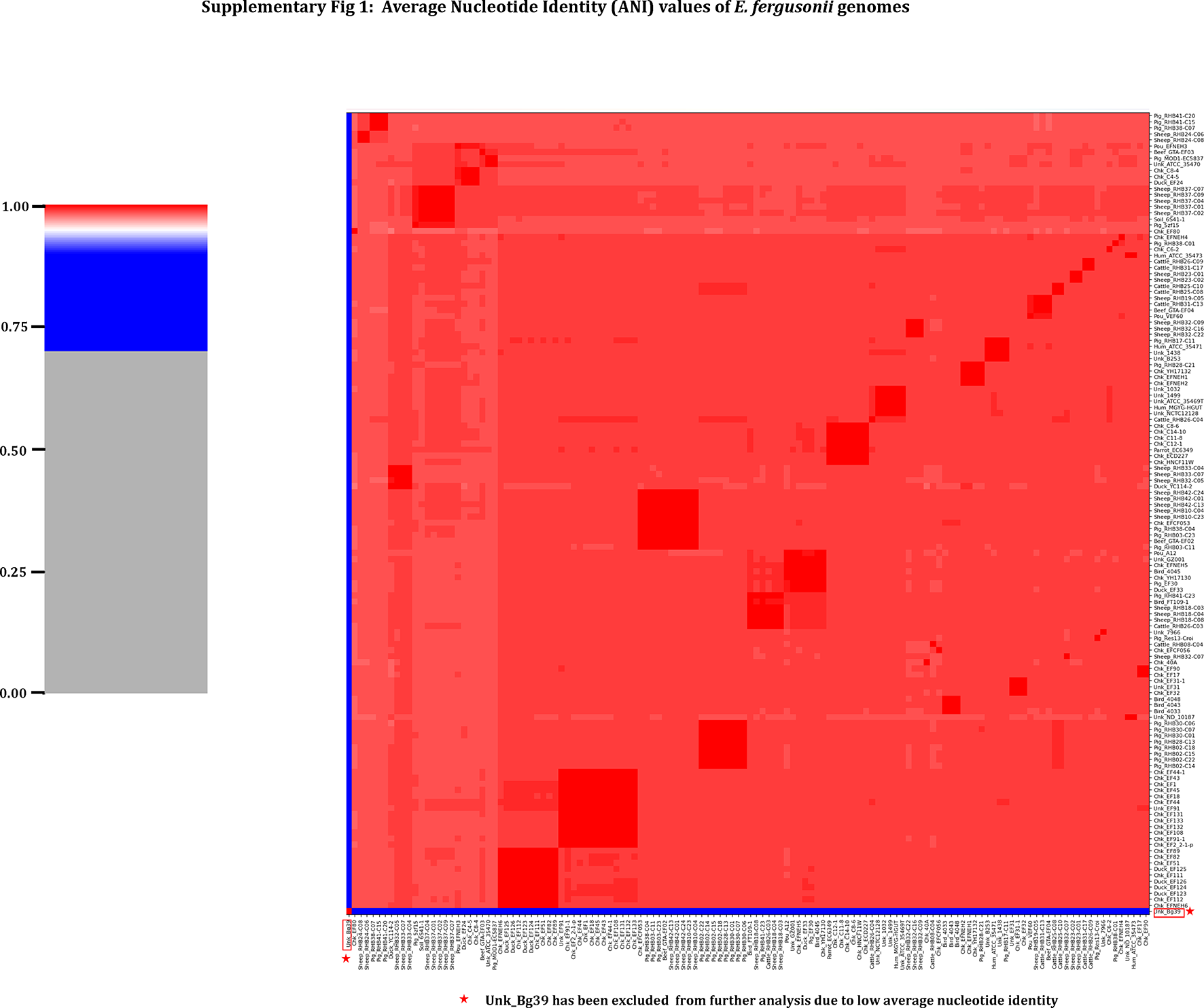

Supplement: Supplementary file 1 [file Image_1.TIF]

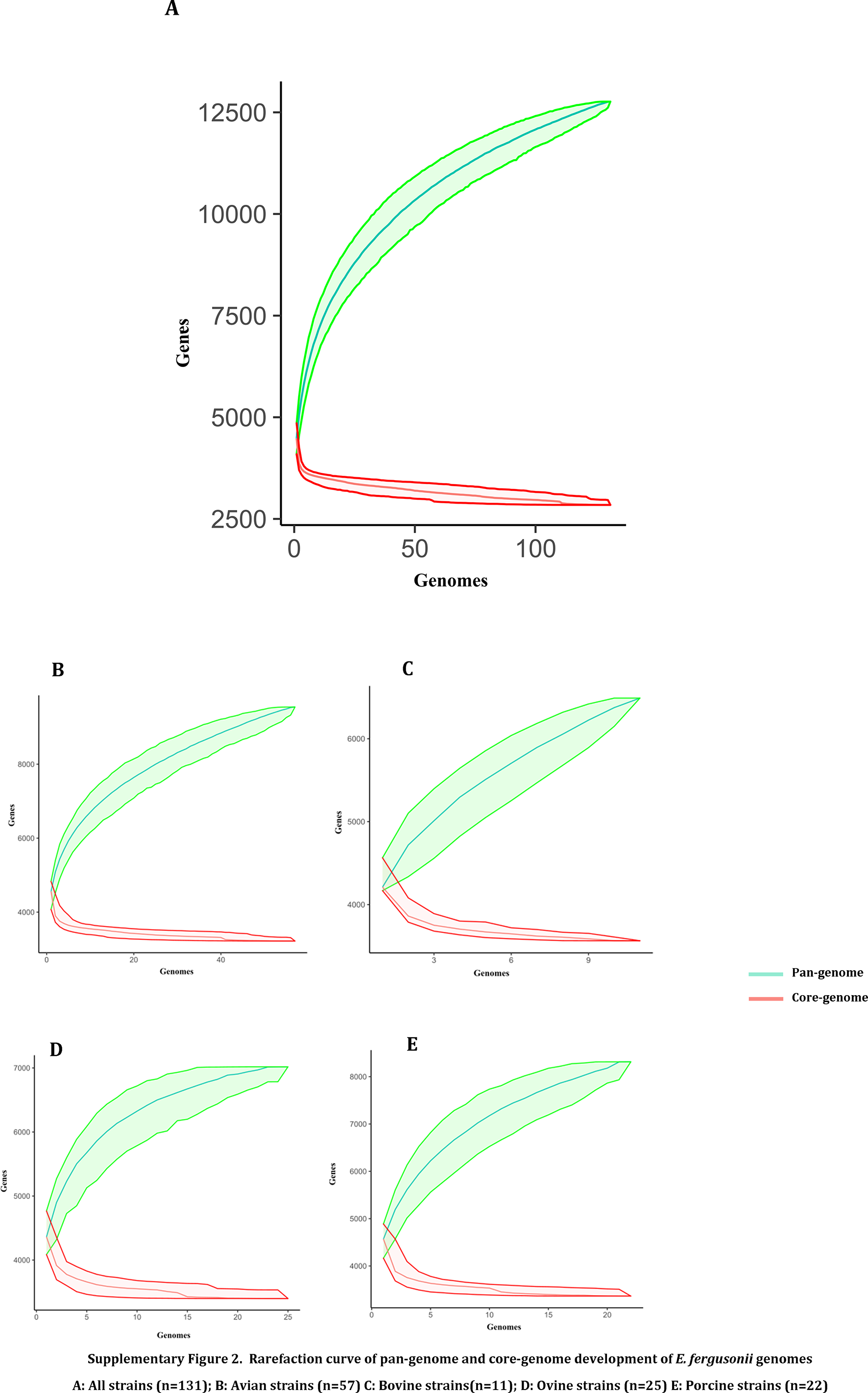

Supplement: Supplementary file 2 [file Image_2.TIF]
